# Single cell transcriptomic analysis reveals transcriptome differences of different cells between eosinophilic chronic rhinosinusitis with nasal polyps and non-eosinophilic chronic rhinosinusitis with nasal polyps

**DOI:** 10.1371/journal.pone.0328241

**Published:** 2025-07-28

**Authors:** Ying Jin, Yuanshan Liang, Zhijun Wang, Yiting Jiang, Fayang Yuan, Tian Zhang

**Affiliations:** Department of Otorhinolaryngology Head and Neck Surgery, Affiliated Hospital of Guizhou Medical University, Guiyang, China; Shanghai 9th Peoples Hospital Affiliated to Shanghai Jiaotong University School of Medicine Department of Plastic Surgery, CHINA

## Abstract

**Background:**

Chronic rhinosinusitis (CRS) with nasal polyps is a heterogeneous chronic inflammatory disease generally divided into two phenotypes including eosinophilic CRS with nasal polyps (eCRSwNP) and non-eosinophilic CRS with nasal polyps (neCRSwNP). However, its pathogenesis remains largely unknown. The aim of this study was to explore mechanistic differences between eCRSwNP and neCRSwNP using a bioinformatics approach.

**Methods:**

We comprehensively analyzed single-cell RNA sequencing data from 3 healthy controls and 6 patients with CRSwNP (including 3 with eCRSwNP and 3 with neCRSwNP) to explore the heterogeneity and potential mechanisms of CRSwNP.

**Results:**

Cluster analysis based on differential gene expression delineated 14 cell clusters. The eCRSwNP group exhibited a markedly higher prevalence of glandular cells and a notable reduction in fibroblasts, myoepithelial cells, and secretory cells compared to patients with neCRSwNP. Functional enrichment analysis of differentially expressed genes revealed the activation of pathways such as IL2-STAT5 signaling and the inhibition of apoptotic pathways in eCRSwNP compared to neCRSwNP. Significant differences in the metabolic profiles of epithelial cell subpopulations were observed between eCRSwNP and neCRSwNP. Furthermore, there were notable discrepancies in the numbers and functionality of immune cells between eCRSwNP and neCRSwNP. The CD4^+^Th2 cell subsets were found to be significantly enriched in eCRSwNP. The highest number of cellular communications from type 2 conventional dendritic cells (cDC2) to CD4^+^Th2 cells was found in CRSwNP, where the ICAM1-CD226 pathway from cDC2 to CD4^+^Th2 was significantly upregulated in eCRSwNP. In addition, eCRSwNP was mainly infiltrated with tissue-resident macrophages, whereas neCRSwNP was mainly infiltrated with monocyte-derived macrophages.

**Conclusions:**

Our study provides new insights into the heterogeneity, molecular mechanisms, and biomarkers of CRSwNP, contributing to improved diagnostic and therapeutic options for this condition.

## 1. Introduction

Chronic rhinosinusitis (CRS) is characterized by persistent nasal/sinus mucosa inflammation for at least 12 weeks without full resolution [1]. Its primary symptoms, including nasal congestion, anterior or posterior rhinorrhea, altered sense of olfaction, and facial pain/pressure, significantly impact patients’ quality of life [2]. CRS is highly prevalent, affecting approximately 12% of adults in the United States and 11% in Europe [3]. In China, the prevalence of CRS is estimated to be 8%, based on face-to-face interviews [4]. CRS is a diverse disease typically classified into two categories: CRS with nasal polyps (CRSwNP) and CRS without nasal polyps (CRSsNP) [5]. Studies have indicated variations in immune responses among CRSwNP patients across different geographical regions and populations with distinct racial backgrounds [6]. This diversity allows for the subclassification of CRSwNP into two distinct endotypes: eosinophilic CRSwNP (eCRSwNP) and non-eosinophilic CRSwNP (neCRSwNP), based on the extent of eosinophil infiltration in polyp pathological sections [6]. Patients with eCRSwNP often exhibit good steroid responsiveness, olfactory dysfunction, comorbid asthma, and a high recurrence rate post-surgery [7]. Furthermore, there has been a notable increase in eosinophilic inflammation, accompanied by elevated IgE production and Th2 response, in CRSwNP patients over time [8]. Thus, understanding the pathological mechanisms underlying the various CRS phenotypes can inform diagnostic and treatment decisions for CRS.

Nasal epithelial cells (NECs) serve as the first physical barrier of the nasal cavity and participate in the initial stage of the Th2-type inflammatory response, playing a vital role in the occurrence and development of CRS [9]. Driven by Th2-type cytokines, NECs undergo various pathological changes: an increase in the number of basal cells, enhanced mucus production, and increased permeability of the airway epithelial barrier, making it more sensitive to oxidants [10,11]. More importantly, epithelial cells and stroma together form an abnormal immune microenvironment, such as eosinophil chemotaxis and IgE enrichment, which enhances the Th2-type inflammatory response in the nasal mucosa [12]. Mechanisms of immune cellular tolerance, specifically Tregs (CD4^+^ Foxp3^+^ regulatory T cells), play a pivotal role in mitigating inflammatory reactions [13]. Existing research indicates that compromised Tregs activity is the primary factor leading to a breakdown in self-tolerance, which can trigger the development of CRSwNP [14]. Macrophages play a central role in the inflammatory process, capable of phagocytosing damaged cells, pathogens, and other apoptotic cells [15]. During this process, these cells transition from being M1-type, which secrete pro-inflammatory cytokines, to M2-type, which possess anti-inflammatory functions. In the development of CRS, both M1 and M2 macrophage counts increase, with a significant rise in M2 macrophages particularly in the later stages, indicating a shift from a pro-inflammatory to an anti-inflammatory role of macrophages [16]. NECs related to the epithelial barrier, as well as immune cells associated with immunity and inflammation, such as CD4^+^ T cells, DCs, and macrophages, play a crucial role in the pathogenesis of CRS [17]. However, the underlying mechanisms are not fully understood and require further investigation.

Cells are the basic units that constitute human tissues and organs. Traditional transcriptome sequencing at the tissue level is a study of cell aggregation, reflecting the average level of a group of cells. However, there are vast differences in the information contained between cells and their expression levels. The heterogeneity of cells can objectively reflect the status information of the occurrence and development of diseases [18,19]. Single-cell RNA sequencing (scRNA-seq) sequences the transcriptome of every single cell in a tissue, enabling the precise identification of cell subtypes, elucidating the specific gene expression of each cell type, analyzing cell development and differentiation trajectories, expounding the interaction and regulatory networks between different cell populations, and systematically depicting the cellular landscape in the immune microenvironment [20]. It has significant advantages in the study of heterogeneous diseases. Therefore, scRNA-seq has been widely used to detect gene expression in different types of cells during reproduction, development, and respiratory inflammatory diseases, thereby revealing the molecular mechanisms of the functions and roles of different cells in these processes [21–26].

In terms of pathogenic mechanisms, the occurrence of events such as epithelial barrier damage, mucus clearance disorders, and immune and inflammatory abnormalities are important reasons for the development and progression of CRS [1]. Abnormalities in cell metabolism, ferroptosis, cell-cell interactions, and cell differentiation may also play a role in the CRS mechanism [27–29]. Different types of cells (mainly epithelial cells and immune cells) play an important role in the pathogenesis of CRS [16,17]. However, the expression characteristics and specific functions of these different cells still need further investigation. There have been some studies using scRNA-seq technology to investigate specific cell-specific gene expression profiles during the development and treatment of CRS [30,31]. These scRNA-seq datasets are helpful for a deeper understanding of the pathogenesis of CRS and are a rich resource for developing novel targeted therapeutic strategies. Our study aims to reveal the transcriptome characteristics of different types of cells in the nasal mucosa of CRS patients, as well as unique mechanisms related to metabolism, ferroptosis, cell-cell interactions, and cell differentiation based on scRNA-seq analysis. This will further elucidate the potential key roles of various cells in the development and progression of CRS.

## 2. Results

### 2.1. Differences in cellular component of the nasal mucosa from different types of CRS patients and healthy donors

**Fig 1:**
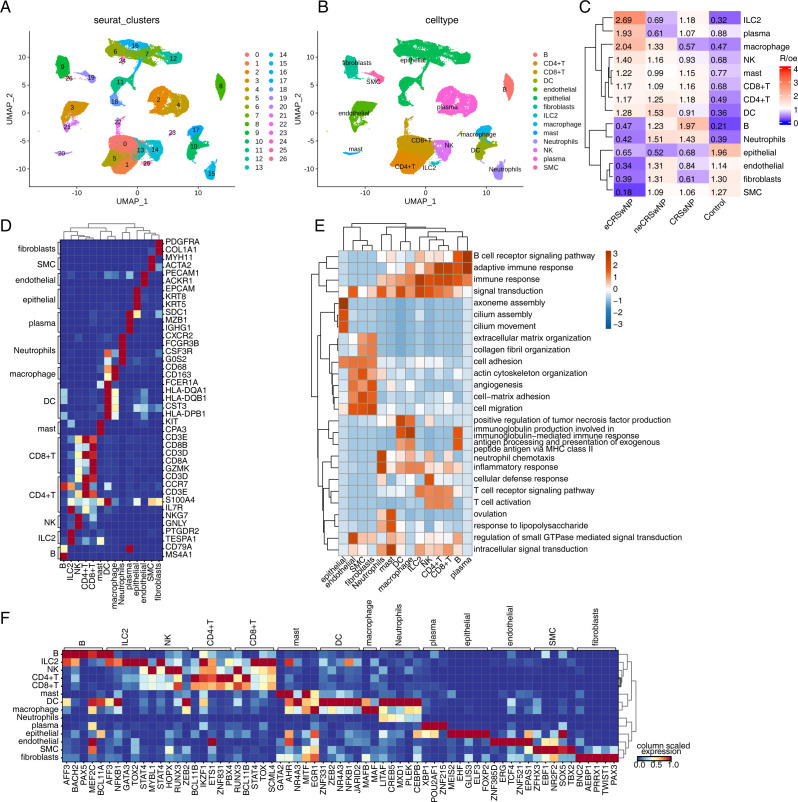
Single-cell transcriptome landscape of human nasal mucosa in control and CRS. (A-B) UMAP plot of single-cell transcriptome profiles. Colors indicate clusters (A) and cell types (B). (C) Tissue prevalence of major cell clusters estimated by Ro/e score. (D) Heat map plot of marker genes in each cell types. (E) Heat map plot showing the most enrichment GO biological process terms of marker genes in each cell types. (F) Heat map plot of top5 transcription factors in each cell types.

We collected published scRNA-seq data from healthy individuals and patients with different types of CRS to analyze the transcriptome expression patterns of the different cells, including nasal mucosa samples from three healthy controls (Control), three CRSsNP, three neCRSwNP, and three eCRSwNP. After strict data quality control, the transcriptome profiling data of 77697 single cells were obtained. The transcriptome expression matrices of these cells were normalized, analyzed by principal component dimensionality reduction, and the top 50 principal components were selected for UMAP dimensionality reduction and visualization; after unbiased clustering analysis, we obtained 27 clusters (Fig 1A). Based on the characteristically expressed genes of each cluster, combined with the marker gene database of reported cells [30], 14 different cell types were identified, including B cells, CD4^+^ T cells, CD8^+^ T cells, dendritic cells (DC), endothelial cells, epithelial cells, fibroblasts, innate lymphocytes 2 (ILC2), macrophages, mast cells, neutrophils, natural killer (NK) cells, plasma cells, and smooth muscle cells (SMC) (Fig 1B).

The enrichment of each cell type in each group was analyzed. Compared with the control group, there was a significant increase in cells such as CD4^+^ T cells, CD8^+^ T cells, and DCs, and a significant decrease in cells such as epithelial cells and SMCs in the CRS group. When comparing eCRSwNP with neCRSwNP, there was a significant increase in cells such as ILC2 cells, macrophages, and mast cells, and a significant decrease in cells such as B cells, endothelial cells, fibroblasts, and neutrophils (Fig 1C). A heatmap showed the relative expression of annotated marker genes for each cell type is presented in Fig 1D. GO enrichment analysis revealed several pathway enrichments: specific genes in epithelial cells were enriched in pathways such as gene filament assembly, cilium assembly, and cilium movement; specific genes in DCs were enriched in pathways such as immune response, inflammatory response, and positively regulated tumor necrosis factor production; specific genes in macrophages were enriched in pathways involved in immunoglobulin production for immune response, neutrophil chemotaxis, and inflammatory response; and CD4^+^ T cells were enriched in pathways such as adaptive immune response, immune response, and T cell activation (Fig 1E).

The top 5 transcription factor genes specifically expressed by each cell cluster were shown in Fig 1F. To analyze the transcriptome changes of CRS at an overall level, we compared the differences between CRSsNP and the control group, as well as between eCRSwNP and neCRSwNP. The analysis results showed that CRSsNP had 532 upregulated genes and 238 downregulated genes compared to the control group, while eCRSwNP had 158 upregulated genes and 67 downregulated genes compared to neCRSwNP (S1A Fig). Through GSEA enrichment analysis, it was found that, compared to the control group, the CRSsNP group showed activation of signaling pathways such as IL-6-JAK-STAT3 and NF-κB, while pathways like Kras signaling were inhibited. In comparison, when comparing eCRSwNP with neCRSwNP, the IL2-STAT5 signaling pathway was activated, while pathways related to cell apoptosis were suppressed (S1B-C Fig). We analyzed the differential genes between CRSsNP vs. Control and eCRSwNP vs. neCRSwNP in different cells. It was found that CRSsNP vs. Control had more Differentially Expressed Genes (DEGs) in plasma cells but fewer DEGs in ILC2s; whereas eCRSwNP vs. neCRSwNP had more DEGs in macrophages but fewer DEGs in SMCs (S1D Fig). A heat map plot was used to display the most enrichment GO biological process terms of DEGs in each cell types (S1E-F Fig).

### 2.2. Analysis of metabolic pathway differences in subpopulations of epithelial cells between eCRSwNP and neCRSwNP

**Fig 2:**
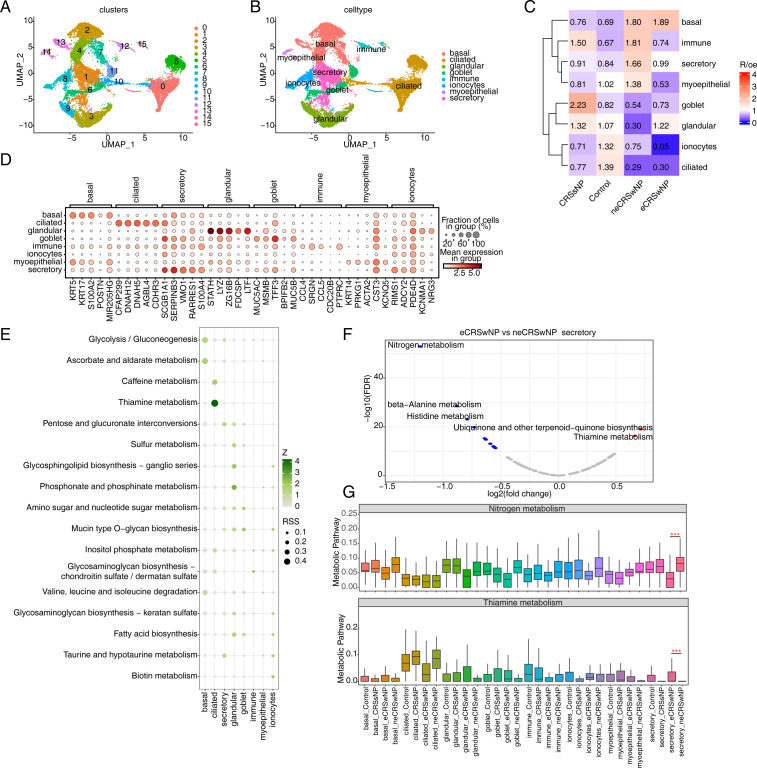
Analysis of metabolic differences in epithelial cell subtypes. (A-B) UMAP plot of epithelial cell subtypes. Colors indicate clusters (A) and cell types (B). (C) Tissue prevalence of major cell clusters estimated by Ro/e score. (D) Dot plot of top5 marker genes in each epithelial cell subtypes. (E) Dot plot showing specific metabolic pathway score in different epithelial cell subtypes. (F) Volcano plot showing the differential metabolic pathways in secretory cells. (G) Box plot showing the score of Nitrongen metabolism and Thiamine metabolism. *** p < 0.001.

All epithelial cells were extracted and clustered to obtain 16 clusters (Fig 2A). These clusters were primarily classified into seven cellular subpopulations based on previously known markers, including goblet cells, basal cells, ciliated cells, submucosal glandular cells, secretory cells, myoepithelial cells, and ionocytes (Fig 2B). According to Ro/e enrichment analysis, CRS showed a significant decrease in ciliated cells compared to control. In addition, patients with eCRSwNP had significantly more glandular cells and significantly fewer myoepithelial and secretory cells compared to patients with neCRSwNP (Fig 2C). Fig 2D illustrated the top 5 marker genes for each cell subpopulation. To explore the differences in epithelial cell metabolism between eCRSwNP and neCRSwNP, we first analyzed the specific metabolic pathways of each epithelial cell subpopulation, with the specific metabolic pathways of basal cells being the glycolysis/gluconeogenesis, ascorbate and aldarate metabolism pathways; those of ciliated cells being the caffeine and thiamine metabolism pathways. Specific metabolic pathways for the glandular cells being the glycosphingolipid biosynthesis-ganglio series and phosphonate and phosphinate metabolism, and for ionocytes were the biotin metabolism pathway (Fig 2E).

The metabolic pathways of secretory cells in the eCRSwNP and neCRSwNP groups were compared. Pathways such as ubiquinone and other terpenoid-quinone biosynthesis and thiamine metabolism were upregulated, whereas pathways such as nitrogen metabolism, β-alanine metabolism, and histidine metabolism were downregulated in eCRSwNP (Fig 2F), with thiamine metabolism pathways upregulated and nitrogen metabolism downregulated in the two epithelial cell subsets, as shown in Fig 2G. We also compared the metabolic pathways of the two groups of cilia, eCRSwNP and neCRSwNP, and found that eCRSwNP exhibited up-regulation of etherolipid metabolism, amino acid sugar and nucleotide sugar metabolism, and nicotinamide and nicotinamide metabolism, while thiamine metabolism and oxidative phosphorylation were downregulated (S2A Fig).

The metabolic pathways of the two groups of cilia, eCRSwNP and neCRSwNP, were compared. The results showed that eCRSwNP exhibited up-regulation of ether lipid metabolism, amino sugar and nucleotide sugar metabolism, and niacin and nicotinamide metabolism, while eCRSwNP exhibited down-regulation of thiamine metabolism and oxidative phosphorylation. The metabolic scores of oxidative phosphorylation and niacin and nicotinamide metabolism pathways in two groups of different epithelial cell subsets were shown in S2B Fig.

### 2.3. Analysis of gene differences related to ferroptosis in CD4+ Th2 cells between eCRSwNP and neCRSwNP

**Fig 3:**
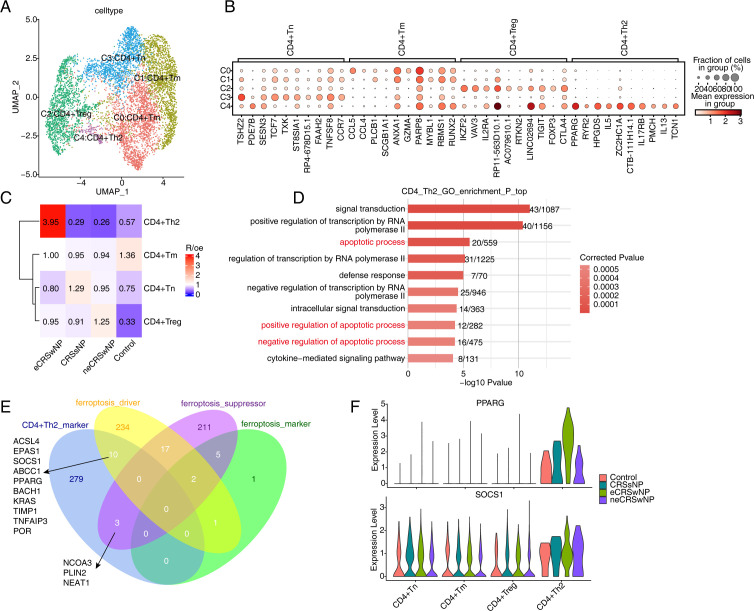
CD4+ Th2 cells are specific in eCRSwNP. (A) UMAP plot of CD4+ T cell subtypes. Colors indicate cell types. (B) Dot plot of top5 marker genes in each CD4+ T cell subtypes. (C) Tissue prevalence of major cell clusters estimated by Ro/e score. (D) Bar plot showing the most enrichment GO biological process terms of marker genes in CD4+ Th2 cells. (E) Venn plot showing the overlapped genes of CD4+ Th2 markers and ferroptosis genes. (F) Violin plot of PPARG and SOCS1 in CD4+ T cell subtypes.

This study focused on transcriptome changes in CD4^+^ T cells. All CD4^+^ T cells were extracted and re-clustered, resulting in five CD4^+^ T cell clusters. These clusters were annotated into four T cell subpopulations, including CD4+ memory T cells (Tm), CD4+ naive T cells (Tn), CD4^+^ regulatory T cells (Treg), and CD4^+^ helper T cells 2 (Th2) (Fig 3A). The bubble chart showed the relative expression of the top 10 marker genes for each CD4^+^ T cell subpopulation, indicating specific expression of these marker genes in different subpopulations (Fig 3B). Based on Ro/e enrichment analysis, compared to the control group, CRS showed a significant decrease in CD4^+^ Tm cells and a marked increase in CD4^+^ Treg cells. In comparison, eCRSwNP demonstrated a notable increase in CD4^+^ Th2 cells compared to neCRSwNP, with CD4^+^ Th2 showing the highest enrichment in eCRSwNP (Figs 3C, S3A). GO enrichment analysis of marker genes in CD4^+^ Th2 revealed that CD4^+^ Th2 is primarily enriched in regulatory pathways related to apoptotic processes (Fig 3D). To explore the mechanisms related to ferroptosis in CD4^+^Th2, we overlapped the marker genes of CD4^+^Th2 with the list of ferroptosis-related genes, resulting in 13 genes, including 10 ferroptosis-driving genes and 3 ferroptosis-suppressing genes (Fig 3E). The figures displayed the expression of ferroptosis-related genes in different CD4+ T cell subpopulations of CD4^+^Th2 (Figs 3F, S3B).

### 2.4. Analysis of differences in intercellular communications between dendritic cells and CD4+ T cells between eCRSwNP and neCRSwNP

**Fig 4:**
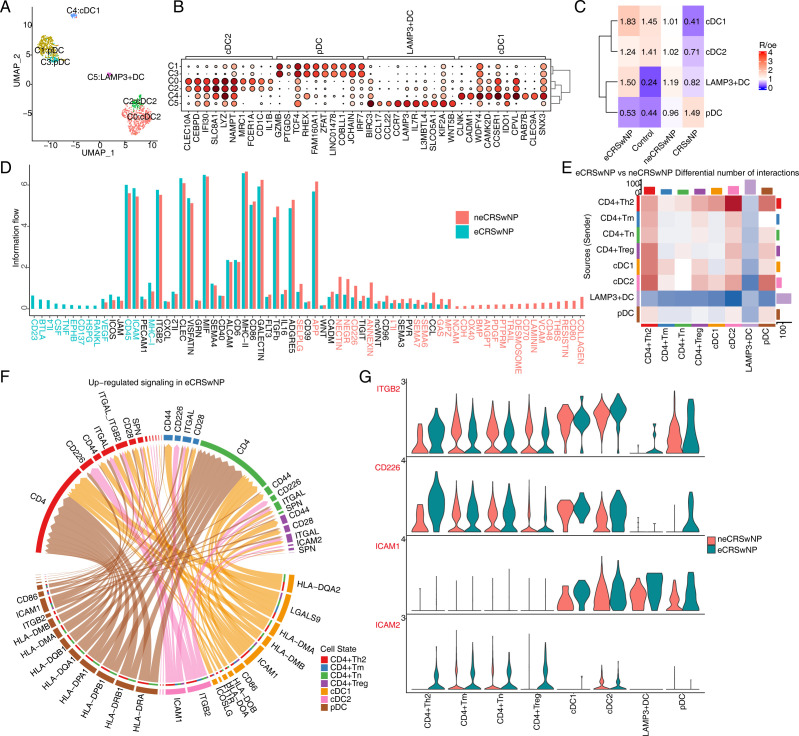
Communication between DCs and CD4+ T cells. (A) UMAP plot of DC cell subtypes. Colors indicate cell types. (B) Dot plot of top10 marker genes in each DC cell subtypes. (C) Tissue prevalence of major cell clusters estimated by Ro/e score. (D) Signaling pathways with significant differences in the overall information flow of each sample groups. (E) Differential number of interactions in DCs and CD4+ T cells. (F) Chord diagram showing up-regulated ligand-receptor pairs in DCs and CD4+ T cells. (G) Violin plot showing expression of ligand-receptor pairs in DCs and CD4+ T cells.

This study extracted all DCs and performed re-clustering, resulting in six DC clusters. These clusters were annotated into four DC subpopulations, including cDC2, plasmacytoid dendritic cells (pDC), cDC1, and LAMP3+ DC (Fig 4A). The bubble chart illustrates the relative expression of the top 10 marker genes for each DC subpopulation, revealing specific expression of these marker genes across different cell clusters (Fig 4B). GO enrichment analysis indicates that the DC subpopulations are primarily enriched in pathways such as positive regulation of I-κB kinase/NF-κB signaling and apoptotic processes (S4 Fig). According to Ro/e enrichment analysis, cDC1 showed a significant increase in eCRSwNP compared to neCRSwNP, while pDC decreases notably (Fig 4C). Additionally, we analyzed the cellular communication between DCs and CD4^+^ T cells in eCRSwNP and neCRSwNP using Cellchat. The figure demonstrates the differences in overall information flow between eCRSwNP and neCRSwNP, with certain signaling pathways enriched in eCRSwNP and others enriched in neCRSwNP (Fig 4D). The heatmap provides an overview of the number of differential interactions between different DC subpopulations and CD4^+^T cell subpopulations, with the highest number of cellular communications observed from cDC2 to CD4^+^ Th2 cells (Fig 4E).

GO enrichment analysis revealed that DC subpopulations are primarily enriched in pathways such as positive regulation of I-κB kinase/NF-κB signaling and apoptotic processes (S4A Fig). The heatmap provides a comprehensive view of the number of differential interactions between various DC subpopulations and CD4^+^ T cell subpopulations, with the highest number of cellular communications observed from cDC2 to CD4^+^ Th2 cells (Fig 4E). We examined the receptor-ligand signaling pathways that differ between eCRSwNP and neCRSwNP. Among the upregulated signals in eCRSwNP are ligands such as CD86, ICAM1, ITGB2, and HLA-GMB from the pDC subpopulation targeting the CD4 receptor in CD4^+^ Th2 and CD4^+^ Tn cell subpopulations. Ligands ICAM1 and ITGB2 from the cDC2 subpopulation target multiple CD4^+^ T cell subpopulations, while ligands ICOSLG, F11R, HLA-DOA, and HLA-DOB from the cDC1 subpopulation target various CD4^+^ T cell subpopulations (Fig 4F). The expression of relevant receptors and ligands involved in cDC2 to CD4+ Th2 cell communication is illustrated in the figure (Fig 4G). Downregulated signals in eCRSwNP include the APP ligand from the pDC subpopulation targeting the CD74 receptor in multiple CD4+ T cell subpopulations, ligands TNFSF4, CD80, CD70, and CD86 from the LAMP3+ DC subpopulation targeting various CD4^+^ T cell subpopulations, and ligands IL17, CD86, and APP from the cDC2 subpopulation targeting multiple CD4^+^ T cell subpopulations. Additionally, the CADM1 ligand from the cDC1 subpopulation targets the cDC1 subpopulation itself (S4B Fig).

### 2.5. Analysis of differences in developmental trajectory of macrophages between eCRSwNP and neCRSwNP

**Fig 5:**
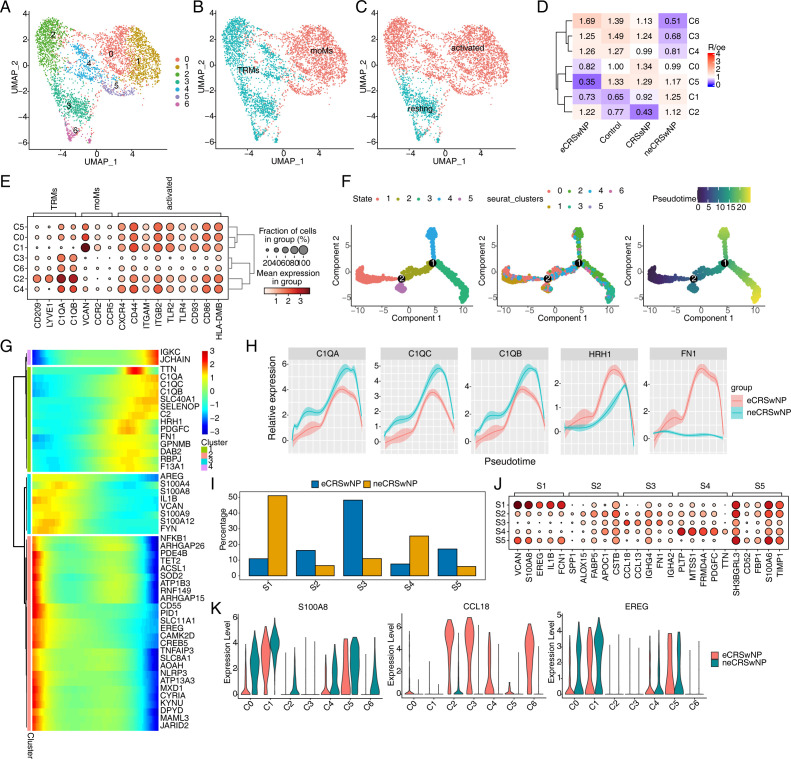
Developmental trajectory of macrophages. (A-C) UMAP plot of macrophage subtypes. Colors indicate clusters and cell types. (D) Tissue prevalence of major cell clusters estimated by Ro/e score. (E) Dot plot of marker genes in each macrophage subtypes. (F) Pseudo-time plots of macrophage. Color-coded according to states, clusters and pseudo-time value. (G) Heat map showing of differential pseudo-time genes. (H) Line chart showing the C1QA, C1QB, C1QC, HRH1 and FN1 relative expression according by pseudo-time. (I) Bar plot of pseudo-time states in different groups. (J) Dot plot of top5 marker genes in each pseudo-time states. (K) Violin plot of S100A8, CCL18 and EGER in macrophage subtypes.

All macrophages in the dataset were re-clustered, resulting in a total of seven distinct macrophage clusters (Fig 5A). These clusters were annotated based on relevant macrophage markers. The results indicated that clusters 3 and 6 were predominantly composed of resting tissue-resident macrophages (TRMs), whereas clusters 2 and 4 were predominantly activated TRMs. Clusters 0 and 1 were mainly composed of activated monocyte-derived macrophages (moMs) (Fig 5B-C).

In addition, scatter plots were used to display DEGs between moMs and TRMs, with particular emphasis on CCL18 and CCL24 (S5A-B Fig). Analysis using the Ro/e formula revealed that clusters 3, 4, and 6 of macrophages were significantly upregulated in eCRSwNP compared to neCRSwNP, whereas clusters 1 and 5 were significantly downregulated (Fig 5D). Bubble plots illustrated the relative expression of annotated marker genes across different macrophage clusters, demonstrating the specific expression of these markers within each cluster (Fig 5E).

To gain a deeper understanding of the differentiation and gene expression patterns of macrophages in CRS, we performed a pseudotime trajectory analysis of macrophages. The analysis identified six pseudotime state branches (S1-S6), with the main transition from clusters 1, 0, and 5 to clusters 2, 3, and 6, indicating a differentiation from moMs to TRMs (Fig 5F). Heatmaps depicted the dynamic expression changes of macrophage genes along the pseudotime trajectory, with C1QA and SLC40A1 showing a gradual increase, whereas S100A8 and S100A9 showed a decrease. TET2 and SLC11A1 showed significant expression differences at the beginning and end of the pseudotime, which may be related to the differentiation process of macrophages. Among these genes, C1QA, C1QB, C1QC, HRH1, and FN1 showed differential expression between eCRSwNP and neCRSwNP during pseudotime analysis (Fig 5H). Notably, the expression of C1QA, C1QB, and C1QC was consistently lower in eCRSwNP than in neCRSwNP, genes associated with M2 polarization of CRSwNP macrophages, suggesting a closer relationship between neCRSwNP and M2 macrophage polarization. The proportion of pseudo-time state branches in the two groups indicated that the S3 state branch was significantly higher in eCRSwNP, whereas the S1 state branch was significantly lower, suggesting that eCRSwNP may be predominantly TRMs, whereas neCRSwNP may be predominantly moMs (Fig 5I). Bubble plots showed the relative expression of the top five marker genes in each pseudotime state branch (Fig 5J), with S100A8 and EREG in S1 and CCL18 in S3 of particular interest, as their expression may contribute to the progression of CRS (Fig 5K).

## 3. Materials and methods

### 3.1. Retrieval and process of public data

Raw data of single-cell RNA-seq data were downloaded from the Genome Sequence Archive (https://ngdc.cncb.ac.cn/gsa-human/browse/HRA000772). The cellranger (v 7.0.0) secondary analysis pipeline was used to generate a digital gene expression matrix. The UMI count matrix was converted into a Seurat object by the R package Seurat [32] (version 4.3.0). Cells with UMI numbers <1000 or with detected genes < 300 or with over 15% mitochondrial-derived UMI counts were considered low-quality cells and were removed. Genes detected in less than 3 cells were removed for downstream analyses.

### 3.2. scRNA-seq data preprocessing

After quality control, the UMI count matrix was log normalized. Then top 2000 variable genes were used to create potential Anchors with FindIntegrationAnchors function of Seurat. Subsequently, IntegrateData function was used to integrate data. To reduce the dimensionality of the scRNA-Seq dataset, principal component analysis (PCA) was performed on an integrated data matrix [33]. With Elbowplot function of Seurat, top 50 PCs were used to perform the downstream analysis. The main cell clusters were identified with the FindClusters function offered by Seurat, with resolution set as default (res = 0.6). And then they were visualized with tSNE or UMAP plots. To identify the cell type for each cluster, we detected gene markers for each cell clusters using the “FindMarkers” function in Seurat package, then we annotated cell types using ScType tools [34].

### 3.3. Differential gene expression analysis

DEGs were determined with the FindMarkers/ FindAllMarkers function from the Seurat package (one-tailed Wilcoxon rank sum test, p values adjusted for multiple testing using the Bonferroni correction). For computing DEGs, all genes were probed that the expression difference on a natural log scale was at least 0.5 and adjusted p value was less than 0.05.

### 3.4. Pseudotime analysis

Monocle2 (version 2.26.0) [35],was used for pseudotime analysis, trajectory construction, and calculation of pseudotime-dependent gene expression. DDRTree (version 0.1.5) method is used for dimension reduction. Cell trajectory was visualized according to cell subtypes and cell states. The Basic Differential Analysis algorithm is used to identify differently expressed gene according to pseudotime function. BEAM (Branched Expression Analysis Modeling) was used to identify genes that are regulated in a branching-dependent manner.

### 3.5. Cell–cell communication

Intercellular communication is essential for coordinating the behavior of individual cells, and it frequently induces changes in cellular state and function [36]. Cell–cell interactions based on the expression of known ligand–receptor pairs in different cell types were inferred using CellChat (v1.6.1) [37]. To identify potential cell–cell communication networks perturbed or induced, we followed the official workflow and loaded the normalized counts into CellChat and applied the preprocessing functions identify Over Expressed Genes and project Data with standard parameters set. As database, we selected the Secreted Signaling pathways and used the precompiled mouse Protein–protein-Interactions as a priori network information. For the main analyses the core functions computeCommunProb, computeCommunProbPathway and aggregateNet were applied using standard parameters and fixed randomization seeds. Finally, to determine the senders and receivers in the network, the function net Analysis_signallingRole was applied on the netP data slot.

### 3.6. Tissue enrichment analysis

The enrichment analysis methodology referenced previous studies [38,39]. To quantify the enrichment of cell types across different tissues, we compared the observed and expected cell numbers for each cluster in each tissue according to the following formula, Ro/e=(observed/expected), where the expected cell numbers of cell clusters in a given tissue were calculated from the Chi-square test [40]. We assumed that one cluster was enriched in a specific tissue if Ro/e > 1.

### 3.7. Other statistical analysis

The scMetabolism [41] algorithm was used for single-cell metabolic analysis. Ferroptosis gene was from FerrDb V2 database [42]. The pheatmap (v1.0.12) package in R was used to perform the clustering based on Euclidean distance. Principal component analysis (PCA) analysis was performed by R package factoextra (v1.0.7) to show the clustering of samples with the first two components. The common statistical analysis methods referenced previously reported data [43].

## 4. Discussion

ECRSwNP is an endotype of CRS with nasal polyps characterized by more severe symptoms, a stronger association with asthma and a higher risk of recurrence [7]. ECRSwNP represents a more aggressive phenotype characterized by greater disease severity, poorer response to therapeutic interventions and higher polyp recurrence rates after surgery. Therefore, it is crucial to thoroughly investigate the pathogenesis of different phenotypes of CRSwNP.

We first analyzed the cellular subpopulation characteristics of each group, and the results showed that eCRSwNP had a significant increase in cells such as ILC2, macrophages, and mast cells, and a significant decrease in B cells, neutrophils, endothelial cells, and fibroblasts compared with neCRSwNP. Our finding of significant changes in immune cells in CRSwNP suggests that immune dysfunction is intimately involved in the pathogenesis of CRSwNP and that the immunological characteristics of the 2 subtypes, eCRSwNP and neCRSwNP, are markedly different. In addition, we found that fibroblasts differed between the 2 groups. Fibroblasts are the main effector cells promoting fibrosis in CRSwNP, which play an important role in inflammation and nasal mucosal remodeling [44]. eCRSwNP has a reduced number of fibroblasts compared to neCRSwNP, suggesting that eCRSwNP is less fibrotic and has a different pathological profile. GO enrichment analysis of all DEGs of eCRSwNP vs. neCRSwNP revealed that upregulated genes were enriched in the pathways of eosinophil chemotaxis, immune response, and adaptive immune response, and downregulated genes were enriched in the pathways of viral defense, bacterial defense, and antimicrobial humoral response. These results highlight the significant immunological differences between eCRSwNP and neCRSwNP, which may be important for the heterogeneity of CRS. In addition, the results of GSEA suggested that eCRSwNP activated pathways such as IL2-STAT5 signaling while inhibiting apoptotic pathways compared to neCRSwNP. It has been reported that Chinese patients with CRSwNP are immunologically characterized by an increased number of Th17 cells and impaired Treg cell function, and the STAT5-IL-2 pathway may promote the Th17/Treg cell imbalance [45]. Furthermore, the apoptotic pathway was inhibited in eCRSwNP compared to neCRSwNP, suggesting that apoptosis is associated with differences in the pathogenesis of these 2 subtypes. A study comparing differences in cell survival and apoptosis in paranasal sinus mucosa from healthy and CRSwNP patients found significantly increased expression of pro-apoptotic gene markers in patients with CRS [46]. In conclusion, there are significant differences in the proportions of cellular subpopulations between the different types of CRSwNP. These differences may be related to the different immune as well as pathological characteristics between these two subtypes.

Epithelial cells constitute a vital element of the epithelial barrier of the nasal mucosa. Epithelial barrier damage is recognized as a pivotal pathogenic mechanism in CRS with nasal polyps (CRSwNP) [17]. Consequently, our study proceeded to conduct an in-depth analysis of the epithelial cells within the dataset to elucidate their role in the CRSwNP. Compared to normal control, ciliated cells in both neCRSwNP and eCRSwNP were found to be reduced. It has been suggested that there is a significant loss of ciliated cells in CRSwNP, especially eCRSwNP, compared to normal subjects [30]. However, the role of ciliated cells in the difference between the two CRS is not clear. In addition, we observed a decrease in the number of secretory cells in ECRSwNP compared to neCRSwNP. S100A4 is specifically highly expressed in secretory cells. It has been shown that S100A4 expression is increased in the serum of CRSwNP patients [47]. Additionally, S100A4 is involved in the regulation of EMT and promotes nasal mucosal remodeling [48]. The changes in the metabolic pathways in the epithelial cells were further analyzed. The nitrogen metabolism was downregulated and the thiamine metabolism was upregulated in secretory cells in ECRSwNP compared to neCRSwNP. Some studies have reported significantly higher levels of nitric oxide metabolites in the maxillary sinus in patients with CRS, and abnormal levels of nitric oxide and nitric oxide metabolites may damage the sinus epithelium [49]. The downregulation of nitrogen metabolism pathways in secretory cells in eCRSwNP may indicate that the nasal mucosa has a decreased ability to metabolize nitric oxide, which promotes disease progression. Thiamine is a key cofactor in maintaining carbohydrate metabolism and is involved in a variety of important cellular metabolic processes [50]. Upregulation of the thiamine pathway may be an indication of increased secretory cell activity in the eCRSwNP. In addition, thiamine metabolism in ciliated cells was downregulated in eCRSwNP, which may indicate reduced ciliated cell activity. Furthermore, we found enhanced niacin and nicotinamide metabolism and downregulation of oxidative phosphorylation in ciliated cells of eCRSwNP compared to neCRSwNP. Oxidative phosphorylation is known to be an important part of ATP synthesis [51]. Therefore, the energy metabolism of ciliated cells in eCRSwNP may be reduced. In addition, studies have reported that nicotinamide-related compounds are strongly associated with CRS [52,53]. Taken together, altered metabolic pathways in epithelial cell subpopulations may be important for the heterogeneity of CRS.

CD4^+^ T cells, especially Th2 cells, play an important role in the pathogenesis of CRS [9]. Th2 cell-associated inflammatory factors such as PPARG, IL-5, and IL-13 may contribute to the progression of CRS by promoting EMT, mucin overproduction, and type 2 immune responses [9,11]. We further analyzed CD4^+^ T cells and found that the number of Th2 cells was significantly increased in the eCRSwNP group compared with the neCRSwNP group. This result is consistent with previous studies [6,7] and highlights the strong association between eCRSwNP and type 2 immune response. In addition, we found that maker genes in Th2 cells were enriched in apoptosis-related pathways. Ferroptosis, a novel mode of programmed cell death in addition to apoptosis, also plays an important role in CRS [54]. Therefore, we analyzed the overlap of maker genes with ferroptosis related genes in Th2 cells and found that ferroptosis-driving genes SOCS1, PPARγ, TIMP1, TNFAIP3 and ferroptosis-suppressing genes NEAT1 were significantly altered in Th2 cells. It has been reported that these genes may contribute to the development of CRS [55–59].

The potential interactions between CD4^+^ T cells and other cell subsets were further analyzed. It was found that compared to neCRSwNP, the highest difference in the number of cellular communications between cDC2 and CD4^+^ Th2 was found in eCRSwNP. DC is the major antigen presenting cell. It has been shown to influence the progression of CRS by interacting with other immune cells [60]. CRSwNP is known to be mainly associated with the type 2 immune response [11]. cDC1 isolated from neCRSwNP samples can bias naive T (Tn) cells toward Th1 and Th17 phenotypes, whereas DCs in eCRSwNP can bias Tn toward Th2 phenotype [61]. In addition, we found specific high expression of IL1B, CCL17, CCL22, CCR7 and IDO1 in different DC subpopulations. Evidence suggests that IL1B, CCL17, CCL22 and CCR7, which are inflammatory mediators, are closely associated with CRS [62–65], while IDO expression has also been found to be increased in patients with CRS [66]. Further analysis showed a significant upregulation of the ligand molecule ICAM1 on cDC2 for the receptor molecule CD226 on CD4^+^Th2 in eCRSwNP, with possible interactions between the two. The ligand ICAM1 is an adhesion molecule that plays an important role in the intercellular inflammatory cascade [67]. The receptor CD226 is a co-stimulatory molecule associated with inflammatory responses [68]. All this evidence suggests that the ICAM1-CD226 signaling pathway may play an important role in the inflammatory response in CRS.

Macrophages have complex pleiotropic effects on inflammatory responses and tissue remodeling in CRS [16]. Therefore, we investigated the role of different macrophage subpopulations in CRSwNP. The results suggest that TRMs are predominant in eCRSwNP, whereas neCRSwNP may be dominated by moMs. In addition, we found some DEGs such as CCL18 and CCL24 between macrophages in eCRSwNP and neCRS. CCL18 is a chemokine that is induced under inflammatory conditions. Peterson et al. [69] reported significantly elevated levels of CCL18 mRNA in NPs and UTs from CRSwNP patients compared to controls. CCL24 is one of the major chemokines involved in the recruitment of eosinophils into tissues [70]. Some investigators found that CCL24 was highly expressed in patients with nasal polyps [71]. In conclusion, different subtypes of macrophages are intimately involved in the pathogenesis of CRS, but the exact molecular mechanisms remain to be elucidated.

Recent advancements in scRNA-seq have significantly enhanced our understanding of the cellular and molecular heterogeneity in CRSwNP. This study identified 14 distinct cell clusters and revealed significant differences in cellular composition and gene expression profiles between eCRSwNP and neCRSwNP. These findings are in line with previous scRNA-seq studies that have also highlighted the complexity of cellular interactions and inflammatory pathways in CRSwNP. For instance, Wang et al. [30] reported distinct epithelial and fibroblast subtypes in CRSwNP, emphasizing their roles in disease progression. Similarly, Guo et al. [72] identified unique immune cell interactions, such as granzyme K+ CD8+ T cells interacting with fibroblasts, which promote neutrophilic inflammation in CRSwNP. Our study further expands on these findings by identifying specific cell types and pathways that are differentially regulated in eCRSwNP compared to neCRSwNP, such as the enrichment of glandular cells and the upregulation of the IL-2-STAT5 signaling pathway in eCRSwNP.

Moreover, the crosstalk between metabolic pathways and inflammation has emerged as a critical aspect of CRSwNP pathogenesis. Our results showing significant differences in the metabolic profiles of epithelial cell subpopulations between eCRSwNP and neCRSwNP underscore the importance of this interaction. Recent studies have demonstrated that metabolic reprogramming, particularly glycolytic pathways, is associated with tissue remodeling and inflammation in CRSwNP. For example, a study highlighted the role of glycolysis in promoting type 2 inflammation in eCRSwNP [73]. Our findings of activated IL2-STAT5 signaling and inhibited apoptotic pathways in eCRSwNP further support the notion that metabolic changes can drive inflammatory responses. This crosstalk between metabolism and inflammation not only contributes to disease heterogeneity but also presents potential therapeutic targets for CRSwNP.

This study has identified several potential biomarkers that could serve as targets for non-invasive diagnosis and therapeutic interventions in CRSwNP. For example, the significant enrichment of CD4+ Th2 cells and the upregulation of the ICAM1-CD226 pathway in eCRSwNP suggest that these markers could be used to differentiate between eCRSwNP and neCRSwNP in a non-invasive manner [30]. Furthermore, the differential infiltration of tissue-resident macrophages in eCRSwNP and monocyte-derived macrophages in neCRSwNP highlights the potential for developing subtype-specific therapies. These findings are particularly relevant in the context of precision medicine, where targeted therapies based on disease subtypes can improve treatment outcomes and reduce the risk of adverse effects.

The application of these biomarkers in precision medicine could lead to the development of more effective and personalized treatment strategies for CRSwNP. For instance, targeting the ICAM1-CD226 pathway in eCRSwNP could provide a novel therapeutic approach to modulate the type 2 inflammatory response [30]. Similarly, interventions aimed at modulating the metabolic profiles of epithelial cells, as identified in our study, could help in reducing inflammation and tissue remodeling in CRSwNP [74]. Future research should focus on validating these biomarkers in larger cohorts and exploring their potential as therapeutic targets through in vivo and in vitro studies. This approach will not only enhance our understanding of CRSwNP pathogenesis but also pave the way for more effective clinical management of this complex disease.

There are several limitations to this study. Firstly, the number of patients for each CRSwNP subtype is relatively small and needs to be verified in a larger cohort. Secondly, the study only relied on correlated scRNA-seq data, and further validation is required through in vivo and in vitro experiments. Additionally, the lack of longitudinal data limits our ability to explore disease progression or treatment response. Future directions could include multi-omics integration (such as spatial transcriptomics and proteomics) to map the intercellular interactions in the tissue structure, or longitudinal follow-up of patients to verify the stability of biomarkers. Despite these inherent technical defects and limitations, we have reported the cellular and molecular landscape of nasal mucosa tissues at the single-cell level. Our data reveal the complexity of cellular composition and dynamic gene expression alterations in CRSwNP. These findings expand our understanding of the pathophysiological processes of CRSwNP and may provide a comprehensive framework for analyzing disease mechanisms and promote progress in subtype-specific management of CRSwNP.

## 5. Conclusion

Our findings showed that different cell types in the nasal mucosa of CRS had different gene expression characteristics compared with the control group. Abnormal changes in metabolism, ferroptosis, cellular interactions, and other related pathways may be closely related to the development of different types of CRS. In conclusion, this study provides important insights into the molecular mechanisms underlying the pathogenesis of CRSwNP and the heterogeneity among different subtypes.

## Supporting information

S1 Fig Single-cell transcriptome landscape of human nasal mucosa in control and CRS. (A) Bar plot showing the numbers of differentially expressed genes (DEGs) by all cells. (B-C) Bubble plot showing the GSEA enrichment results. (D) Bubble plot showing the numbers of DEGs in different cell types. (E-F) Heat map plot showing the most enrichment GO biological process terms of DEGs in each cell types.
(TIF)

S2 Fig Analysis of metabolic differences in epithelial cell subtypes. (A) Volcano plot showing the differential metabolic pathways in ciliated cells. (B) Box plot showing the score of Oxidative phosphorylation and Nicotinate and nicotinamide metabolism. *** p < 0.001.
(TIF)

S3 Fig CD4+ Th2 cells are specific in eCRSwNP. (A) Bar plot showing the proportion of cells in different groups. (B) Violin plot of TIMP1 and TNFAIP3 in CD4+ T cell subtypes.
(TIF)

S4 Fig Communication between DCs and CD4+ T cells. (A) Heat map plot showing the most enrichment GO biological process terms of marker genes in each DC cell subtypes. (B) Chord diagram showing down-regulated ligand-receptor pairs in DCs and CD4+ T cells.
(TIF)

S5 Fig Developmental trajectory of macrophages. (A-B) Scatter plot showing the top5 DEGs in macrophage subtypes.
(TIF)
