# Supplementary figures and images for: Single cell transcriptomic analysis reveals transcriptome differences of different cells between eosinophilic chronic rhinosinusitis with nasal polyps and non-eosinophilic chronic rhinosinusitis with nasal polyps
Source: PLoS One. 2025 Jul 28;20(7):e0328241. doi: 10.1371/journal.pone.0328241 (PMC12303326; doi:10.1371/journal.pone.0328241)

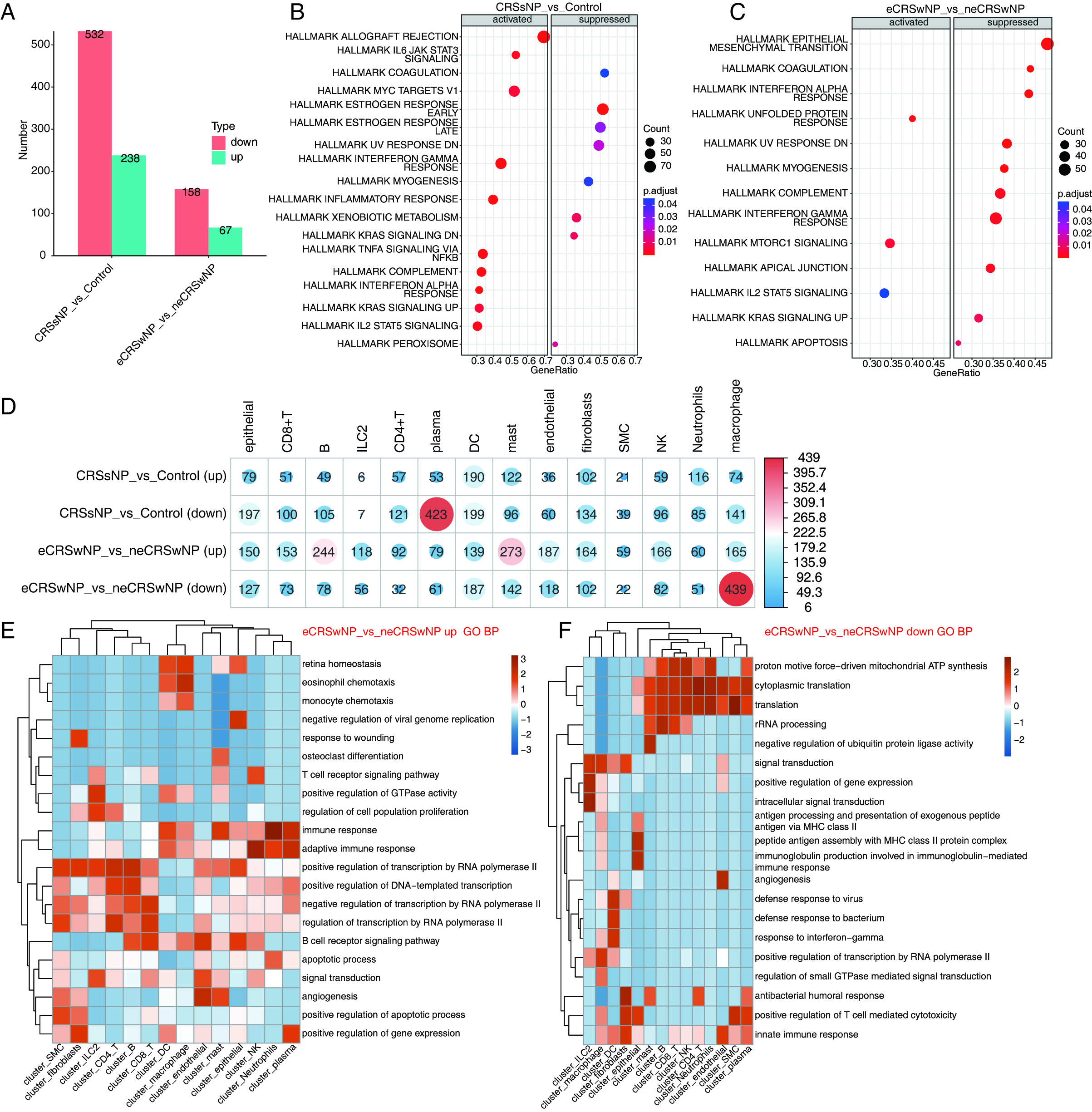

Supplement: S1 Fig — (A) Bar plot showing the numbers of differentially expressed genes (DEGs) by all cells. (B-C) Bubble plot showing the GSEA enrichment results. (D) Bubble plot showing the numbers of DEGs in different cell types. (E-F) Heat map plot showing the most enrichment GO biological process terms of DEGs in each cell types. (TIF) [file pone.0328241.s001.tif]

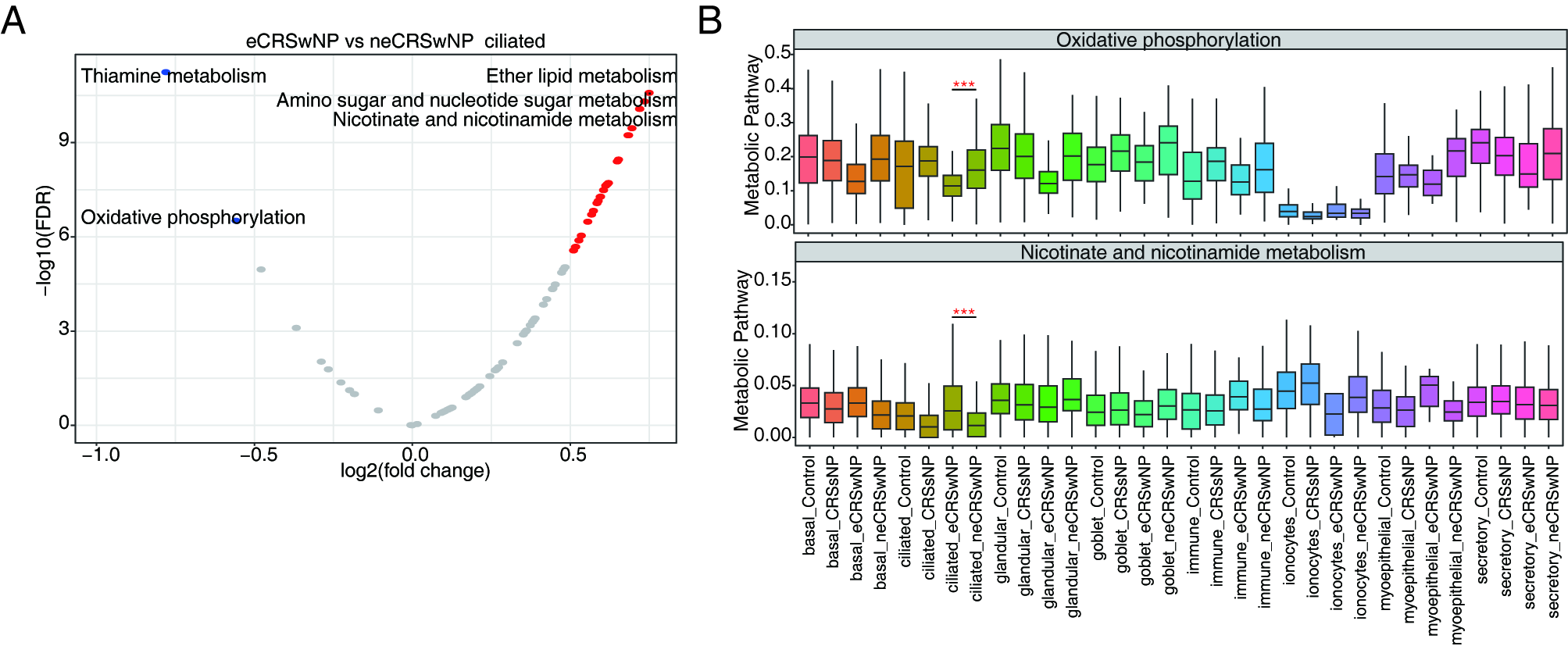

Supplement: S2 Fig — (A) Volcano plot showing the differential metabolic pathways in ciliated cells. (B) Box plot showing the score of Oxidative phosphorylation and Nicotinate and nicotinamide metabolism. *** p < 0.001. (TIF) [file pone.0328241.s002.tif]

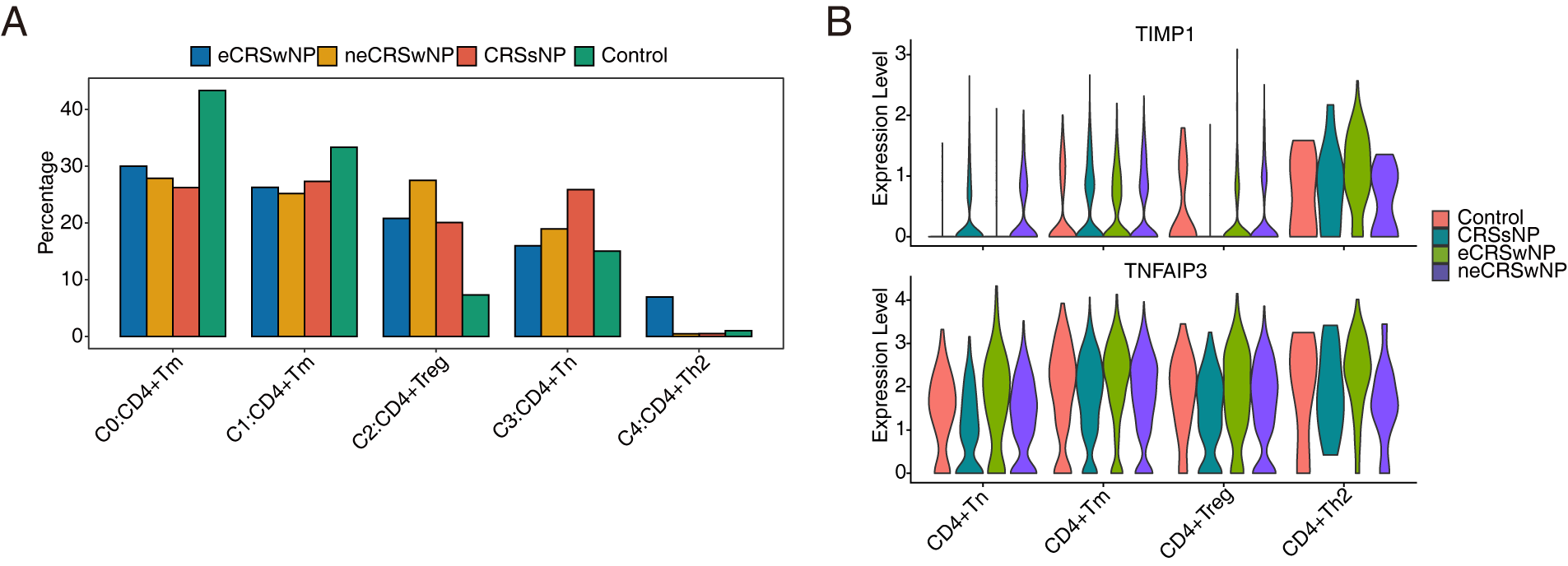

Supplement: S3 Fig — (A) Bar plot showing the proportion of cells in different groups. (B) Violin plot of TIMP1 and TNFAIP3 in CD4+ T cell subtypes. (TIF) [file pone.0328241.s003.tif]

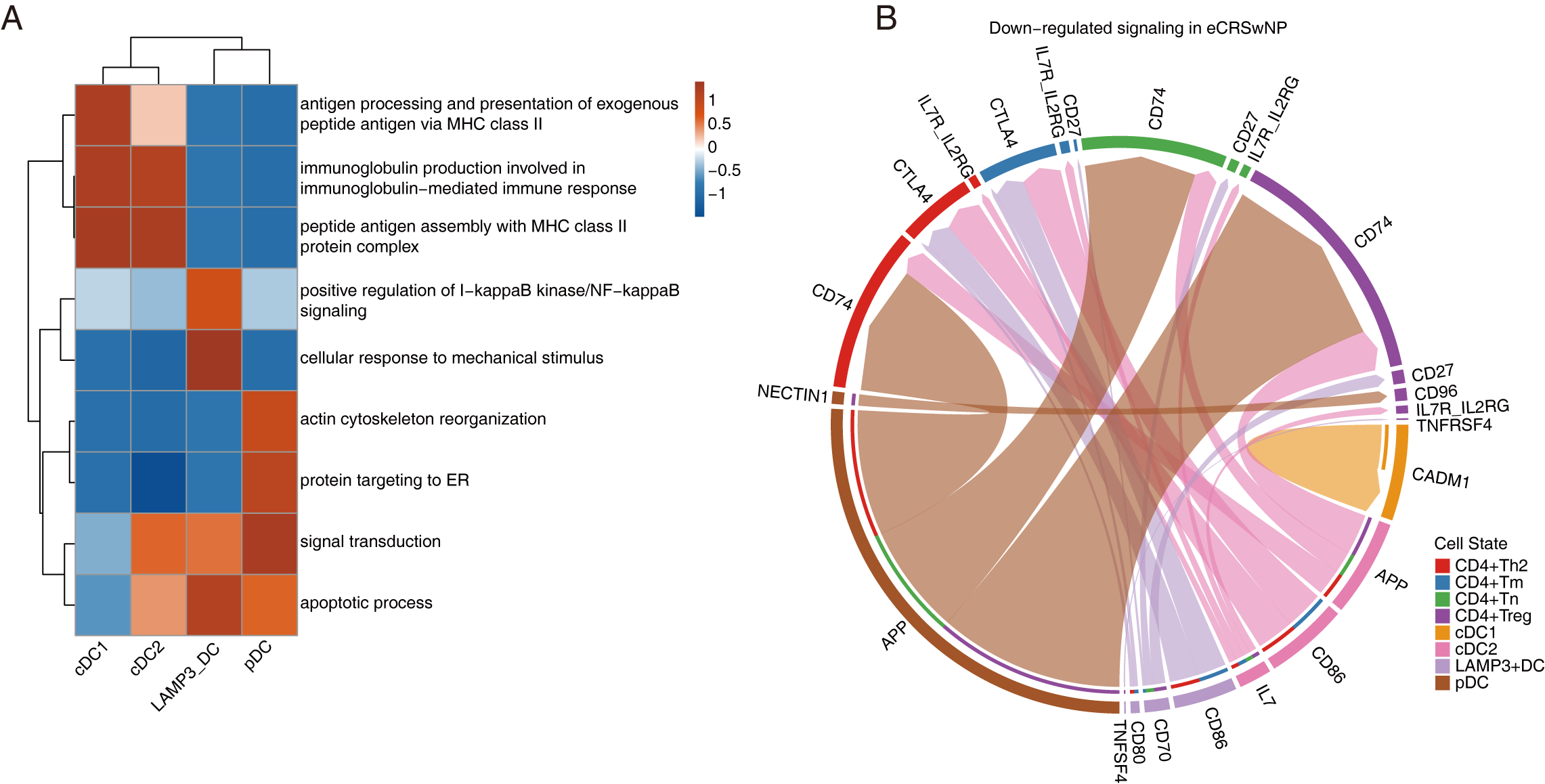

Supplement: S4 Fig — (A) Heat map plot showing the most enrichment GO biological process terms of marker genes in each DC cell subtypes. (B) Chord diagram showing down-regulated ligand-receptor pairs in DCs and CD4+ T cells. (TIF) [file pone.0328241.s004.tif]

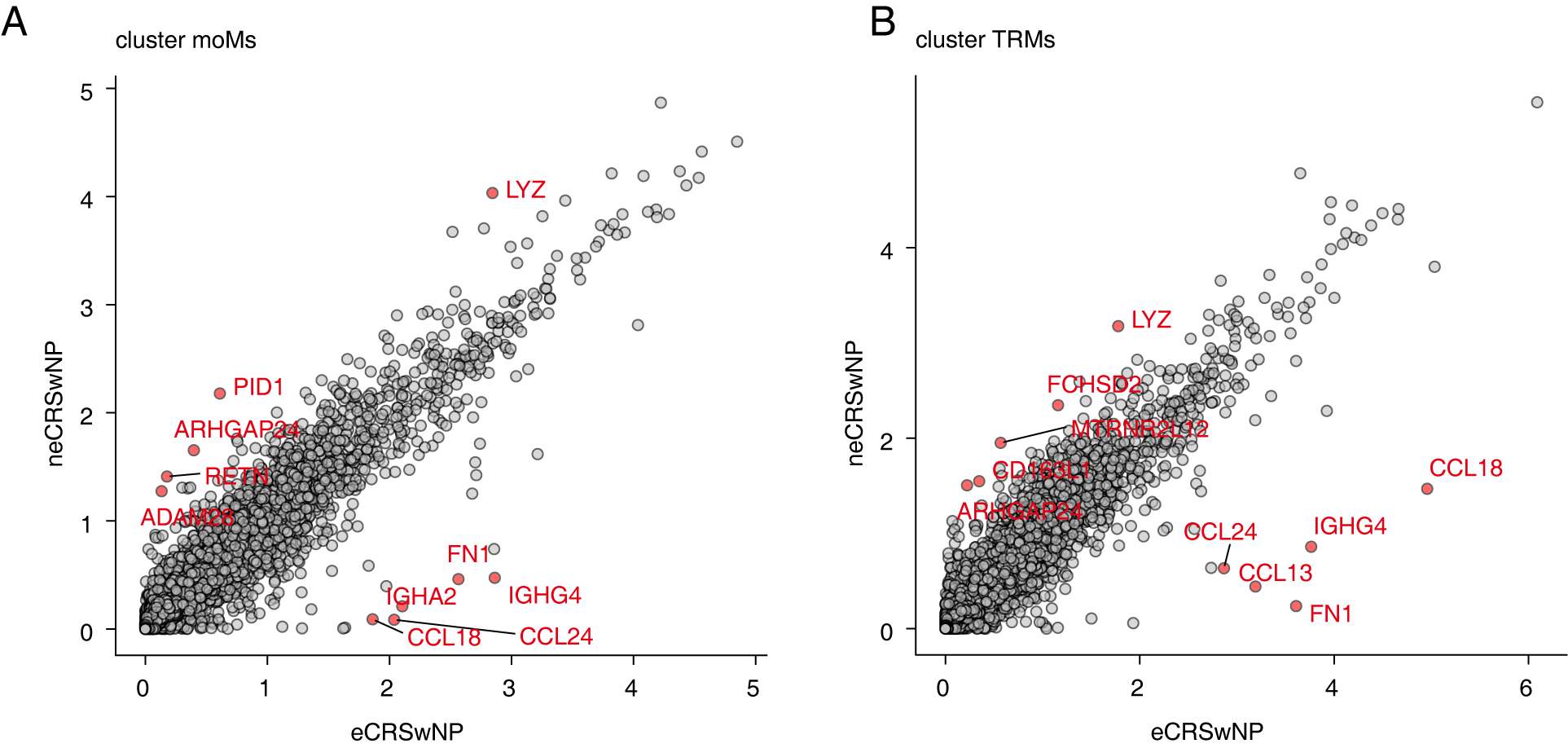

Supplement: S5 Fig — (A-B) Scatter plot showing the top5 DEGs in macrophage subtypes. (TIF) [file pone.0328241.s005.tif]
